# Intermediate Stage of Epileptogenesis Is Not Affected by Disrupting Memory Consolidation Processes

**DOI:** 10.3390/ijms26178364

**Published:** 2025-08-28

**Authors:** Carlos Howey, Autumn McFetridge, Artur Luczak

**Affiliations:** Canadian Centre for Behavioural Neuroscience, University of Lethbridge, 4401 University Drive, Lethbridge, AB T1K 3M4, Canada; chowey@ualberta.ca (C.H.); autumn.mcfetridge@uleth.ca (A.M.)

**Keywords:** epileptogenesis, neurodegeneration, temporal lobe epilepsy, memory consolidation, rapamycin, mTOR

## Abstract

Epilepsy affects 50 to 70 million people worldwide and is characterized by recurring seizures that accelerate neurodegeneration; however, its mechanisms are still not well understood. It was proposed that epileptogenesis may be “hijacking” the mechanisms underlying normal memory formation, where synapses involved in seizure activity are strengthened after each seizure, similarly to the strengthening of memories during the consolidation processes. To investigate this link, we used a kindling model of temporal lobe epilepsy in mice and tested whether disrupting memory consolidation could alter epileptogenesis. Animals were kindled until stage 3 behavioral seizures on the Racine scale were reached. In subsequent sessions, rapamycin was administered within 10 min following kindling to inhibit protein synthesis essential for memory consolidation in the neurons involved in a seizure. Rapamycin reduced the freezing response to sensory stimuli preceding a seizure, suggesting that memory consolidation was disrupted; however, epileptogenesis was not affected. Additionally, we tested whether administering isoflurane, which reduces neuronal activity, could weaken seizure-associated patterns by interfering with memory reconsolidation processes. This intervention also did not reduce the intensity of seizures. Altogether, these preliminary results appear to be inconsistent with the hypothesis that epileptogenesis involves the same mechanisms as memory formation processes.

## 1. Introduction

Epilepsy is a progressive disease characterized by recurrent seizures that affects millions of individuals globally, with pharmacological treatments of epilepsy being purely symptomatic [1], and with temporal lobe epilepsy being one of the most common and treatment-resistant forms of the disease. Epileptogenesis is the process by which normal neuronal circuits transform into hyperexcitable seizure networks, the understanding of which has remained a significant challenge. Recent evidence suggests that epileptogenesis may be “hijacking” the mechanisms behind memory consolidation [2,3,4,5], in which the synaptic connections involved in seizure activity are selectively strengthened. This is similar to the strengthening of synaptic connections as a result of the memory consolidation processes [6]. The search for reliable treatment is necessary, as neurodegeneration in epilepsy occurs after even a single ictal event, which can cause neuronal loss, degeneration, and inflammation in several key brain regions (e.g., hippocampus, striatum, and the amygdala) [7,8]. This neurodegeneration with recurring seizures is thought to be the driving force that lowers seizure thresholds, increasing the frequency and intensity of ictal events in patients with chronic epilepsy [9].

Memory formation involves activity-dependent synaptic plasticity, where synaptic strength is enhanced following repeated neuronal activation [10]. This process is mediated by molecular pathways, such as N-methyl-D-aspartate (NMDA) receptor activation and downstream signaling cascades, which are also implicated in epileptogenesis. For instance, seizures are often associated with pathological high-frequency oscillations (HFOs) that resemble hippocampal sharp wave-ripple events critical for memory consolidation [11]. Moreover, during interictal and ictal periods, neuronal patterns often mimic those seen during spontaneous memory reactivation, particularly during sleep [12,13,14]. Also, in a kindling model of epilepsy, repeated stimulation strengthens synaptic connections, creating lasting hyperexcitability akin to the formation of memory traces [15]. Additionally, many biological compounds like matrix metalloproteinases are involved in both learning and memory processes and epilepsy mechanisms [16]. Moreover, excitation/inhibition balance plays a crucial role in both [17]. Given these parallels, targeting the mechanisms involved in memory consolidation may offer a possible therapeutic strategy for epilepsy. Treatments aimed at disrupting the consolidation of seizure-related synaptic changes could theoretically “de-potentiate” hyperexcitable circuits and mitigate seizure progression. We used rapamycin, an inhibitor of the mammalian target of rapamycin (mTOR) protein, which has been shown to inhibit fear memory reconsolidation [18]. Moreover, we used isoflurane to reduce neuronal activity after a seizure to interfere with the replay of seizure-associated patterns during memory consolidation periods.

In the present study, we tested whether disrupting the memory formation processes could attenuate epileptogenesis in a kindling model of temporal lobe epilepsy by using rapamycin to inhibit synaptic protein synthesis and isoflurane to reduce neuronal reactivation. We found that neither reduced seizure intensity nor progression. These results suggest that, once an intermediate level of seizures is established, post-seizure interference with memory-related plasticity may be insufficient to affect epileptogenesis.

## 2. Results

### 2.1. Rapamycin Experiment

It is well known that kindling is epileptogenic, meaning that with repeated electrical stimulation, epileptic networks expand, encompassing most of the brain while also lowering the seizure threshold (e.g., current); in other words, seizures become more intense and more frequent. The primary goal of this experiment was to test rapamycin as a potential treatment for epilepsy, which would disrupt epileptogenesis when administered following an ictal event. To do this, we first determined the thresholds (minimum current) and suprathresholds (minimum current plus 100 μA) to induce an electrographic seizure (EEG only) for each animal implanted with a bipolar electrode in the amygdala (see Section 4 for details). The animals were kindled every second day until sufficient behavioral seizures (stage 3 on the Racine scale) had manifested (Section 4). For five sessions following the first sufficient behavioral seizure, the experimental animals received an injection of rapamycin (40 mg/kg) intraperitoneally following their removal from the experimental chamber, while the control animals received an injection of saline. To determine any effects of the drug, seizure duration, behavioral seizure intensity (Racine scale), and freezing behavior were all analyzed (Section 4). Additionally, following the treatment sessions, the animals were re-thresholded as an additional measure to determine if the rapamycin had disrupted epileptogenesis.

**Figure 1:**
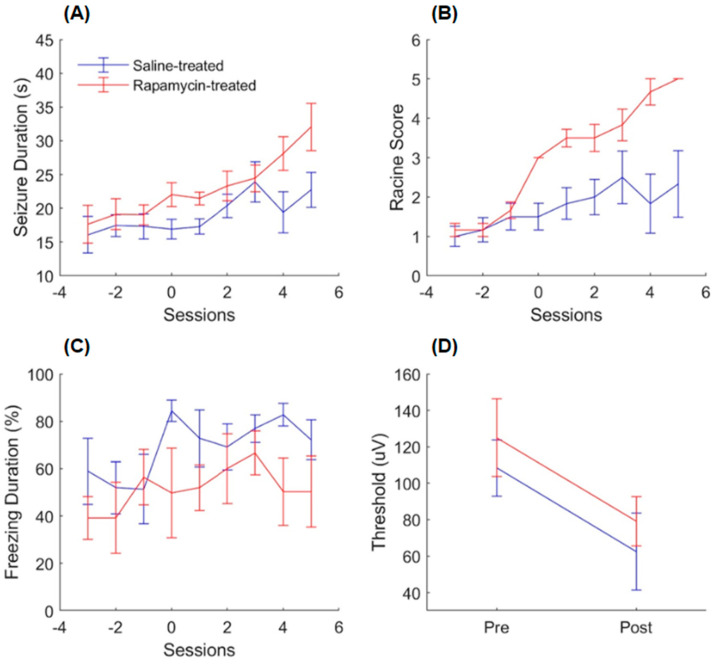
Results of the rapamycin experiment. (**A**) Seizure duration for both control and experimental animals. (**B**) Racine scores for both control and experimental animals. (**C**) Freezing duration for both control and experimental animals. (**D**) Minimum kindling thresholds to evoke seizures for both control and experimental animals. Legend in (**A**) applies to all subfigures. Numbers −3 to −1 indicate kindling sessions; 0 is when behavioral seizures of stage 3 on the Racine scale were identified, and 1 to 5 indicate kindling plus rapamycin sessions. Data reported as mean ± S.E.M; N = 6 in each group.

It was found that rapamycin does not prevent the progression of epilepsy, as the animals treated with rapamycin continued to develop advanced seizures during the treatment sessions. A two-way ANOVA revealed that the rapamycin-treated animals had, on average, seizures that were longer in duration [Figure 1A: main effect of the session, *F*(8, 90) = 4.81, *p* < 0.05; main effect of the treatment, *F*(1, 90) = 14.1, *p* < 0.05; effect of the interaction, *F*(8, 90) = 1.01, *p* > 0.05] and more generalized [Figure 1B: main effect of the session, *F*(8, 90) = 10.5, *p* < 0.05; main effect of the treatment, *F*(1, 90) = 46.4, *p* < 0.05; effect of the interaction, *F*(8, 90) = 3.24, *p* < 0.05]. However, follow-up *t*-tests revealed that the rapamycin-treated animals had seizures that were longer in duration and more generalized even before the treatment sessions had commenced [Figure 1A: session 0, *t*(10) = −2.24, *p* < 0.05; Figure 1B: session 0, *t*(10) = −4.39, *p* < 0.05].

Sensitization is indicative of epileptogenesis and was determined by calculating the difference in the minimum current required to elicit an electrographic seizure prior to and following the experiment [19]. In both groups, sensitization occurred as the average minimum threshold was lowered following the rapamycin experiment in each group. These results are consistent with the behavioral seizure and seizure duration results obtained in the initial analysis (see the above paragraph). A two-way ANOVA revealed that the minimum threshold required to induce a seizure was lowered for both groups following the treatments [Figure 1D: main effect of the session, *F*(1, 20) = 6.34, *p* < 0.05; main effect of the treatment, *F*(1, 20) = 0.840, *p* > 0.05; effect of the interaction, *F*(1, 20) = 0.00, *p* > 0.05]. The average difference between the pre- and post-thresholding results was 45.8 μA for both groups, and a *t*-test found no significant difference [Figure 1D: *t*(10) = 0.00, *p* > 0.05]. These results indicate that sensitization to the convulsive stimulus occurred, and that rapamycin did not disrupt sensitization to the electrical stimulation.

Post-cue freezing was also analyzed, as previous studies have shown that rapamycin is effective in attenuating conditioned responses in a variety of classical conditioning paradigms [18,20,21,22], which provided the framework for the hypothesis that rapamycin may disrupt memory consolidation. Here, the conditioned response is the animal’s freezing in response to the presentation of the conditioned sensory stimuli (auditory and visual). A two-way ANOVA revealed that the rapamycin-treated animals had lower post-cue freezing than the saline-treated animals [Figure 1C: main effect of the session, *F*(8, 90) = 1.11, *p* > 0.05; main effect of the treatment, *F*(1, 90) = 9.97, *p* < 0.05; effect of the interaction, *F*(8, 90) = 0.530, *p* > 0.05].

### 2.2. Isoflurane Experiment

Following the rapamycin experiment, the animals then underwent an additional experiment that tested the effects of isoflurane on the persistence of seizures. This experiment utilized a similar protocol as mentioned above; however, the animals were stimulated with the newly acquired suprathresholds and were placed in an anesthetic induction chamber for 5 min and administered with isoflurane (2.5% in oxygen), following kindling (Section 4). Control animals were placed back into their home cages. It is important to note that the same animals from the previous experiment were utilized and exhibited highly generalized seizures at the start of treatment; thus, this experiment tested the effects of isoflurane on established epileptic networks.

**Figure 2:**
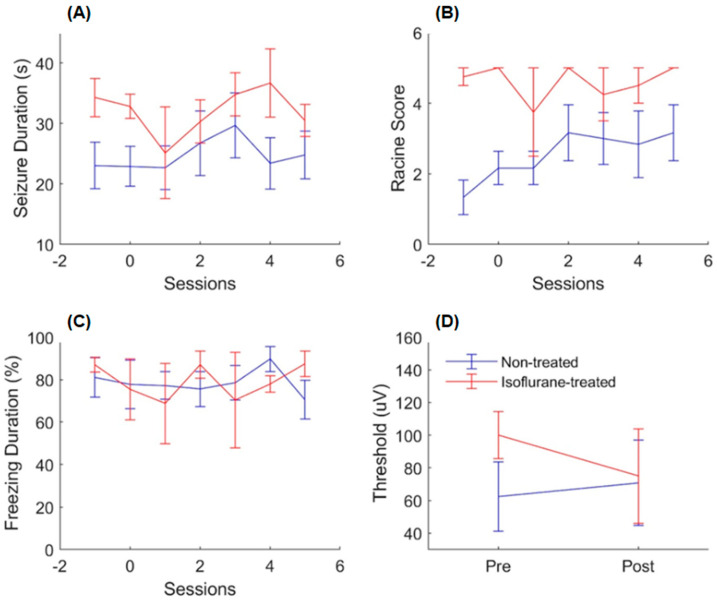
Results of the isoflurane experiment. (**A**) Seizure duration. (**B**) Behavioral seizure score (Racine scale). (**C**) Freezing duration. (**D**) Minimum kindling thresholds to evoke seizures measured before and after the isoflurane experiment. Legend in (**D**) applies to all subfigures. Session 1 in (**A**–**C**) represents the session where isoflurane was first administered. Numbers −1 and 0 indicate kindling sessions; 1 to 5 indicate kindling plus isoflurane sessions. Data reported as mean ± S.E.M.

Using the same analyses as before that measured behavioral seizure intensity and electrophysiological seizure duration, it was found that isoflurane does not disrupt or undo epileptogenesis, as the treated animals continued to display advanced seizures throughout the treatment sessions. A two-way ANOVA revealed that the isoflurane-treated animals displayed, on average, seizures that were longer in duration [Figure 2A: main effect of the session, *F*(6, 56) = 0.630, *p* > 0.05; main effect of the treatment, *F*(1, 56) = 9.19, *p* < 0.05; effect of the interaction, *F*(6, 56) = 0.42, *p* > 0.05] and more generalized [Figure 2B: main effect of the session, *F*(6, 56) = 0.820, *p* > 0.05; main effect of the treatment, *F*(1, 56) = 30.5, *p* = 0.00; effect of the interaction, *F*(1, 56) = 0.610, *p* > 0.05]. No significant effect of the sessions on either independent variable was found, indicating that epileptogenesis had occurred prior to this experiment.

To determine if sensitization occurred, an additional thresholding session was conducted following the isoflurane experiment, which was used to calculate the difference in the minimum threshold to induce an electrographic seizure. A two-way ANOVA revealed that the minimum threshold required to induce a seizure did not change significantly for both groups [Figure 2D: main effect of the session, *F*(1, 14) = 0.03, *p* > 0.05; effect of the treatment, *F*(1, 14) = 0.31, *p* > 0.05; effect of the interaction, *F*(1, 14) = 0.10, *p*> 0.05], which indicates that neither sensitization or tolerance had occurred.

The post-cue freezing analysis was also conducted for this experiment to determine if isoflurane disrupts memory consolidation. A two-way ANOVA revealed no significant difference in post-cue freezing between the isoflurane- and non-treated animals [Figure 2C: main effect of the session, *F*(6, 56) = 0.35, *p* > 0.05; main effect of the treatment, *F*(1, 56) = 0.01, *p* > 0.05; effect of the interaction, *F*(1, 56) = 0.56, *p* > 0.05]. The treated animals displayed typical freezing responses, suggesting that memory consolidation/reconsolidation was not disturbed.

## 3. Discussion

The findings from this study challenge the hypothesis that epileptogenesis “hijacks” the brain’s natural memory formation processes, which suggests that the mechanisms of synaptic plasticity, such as those involved in memory consolidation, are co-opted during seizures to stabilize seizure-related synaptic networks. However, neither rapamycin, an inhibitor of protein synthesis critical for synaptic plasticity, nor isoflurane, which reduces neuronal activity, attenuated seizure progression in our experiments. Together, these findings emphasize the complexity of the relationship between epileptogenesis and memory formation. While similarities in the underlying cellular and molecular mechanisms were first noted by Goddard [23], the failure of rapamycin and isoflurane to disrupt seizure progression challenges the view that epileptogenesis solely relies on “hijacking” a single memory mechanism.

It is important to acknowledge that our results appear to conflict with multiple previous studies that have demonstrated that epileptogenesis and memory consolidation share common mechanisms of synaptic plasticity, particularly within the hippocampus and associated cortical networks, and that epileptiform activity during sleep can impair long-term memory while promoting pathological circuit reorganization (e.g., [3,24,25]). One possible explanation for this discrepancy is that our interventions were applied after seizure networks were already established, whereas previous studies often examined earlier stages when pathological plasticity was still developing.

Our analysis, which measured post-cue freezing, showed that the rapamycin attenuated freezing behavior (Figure 1C). This is consistent with other classical conditioning studies that show a reduction in fear responses after rapamycin application [18,20,21,22]. It has been previously demonstrated that rapamycin readily passes through the blood–brain barrier due to its hydrophobic properties [26] to inhibit the mammalian target of rapamycin (mTOR), which is known to play a critical role in the synaptic plasticity required for memory consolidation and reconsolidation [18,21,27]. mTOR regulates mRNA translation in neurons through the phosphorylation of the p70-kDa ribosomal s6 kinase (p70s6K) and eukaryotic initiation factor 4E-binding protein 1 (4EBP1); in a variety of classical conditioning paradigms, neurons exhibit an increase in phosphorylated p70s6K following training [18,20,21,22]. Rapamycin and the FK506-binding protein (FKBP12) form a protein complex that inhibits mTOR through allosteric regulation [28]. In classical conditioning paradigms, rapamycin has been found to attenuate both the phosphorylation of p70s6K and the conditioned behavior [18,20,21,22]. In cases of tuberous sclerosis complex (TSC), mTOR has been found to play a critical role in the epileptogenesis associated with TSC, as inhibitors of mTOR, such as the drug rapamycin, have been effective in reducing the intensity and frequency of seizures for TSC patients [29]. The results of the rapamycin experiment here show that systemic injections of rapamycin following the recall of a fear memory (sensory stimuli associated with kindling) disrupt the consolidation of that memory, as evidenced by attenuation of the freezing behavior in comparison to the non-treated group. However, considering the lack of effect of rapamycin on seizure intensity, it can be suggested that mTOR signaling is not a universal mechanism required for epileptogenesis in all epileptic conditions and models, but is required for some (e.g., TSC). While mTOR inhibition has shown antiepileptogenic effects in preclinical models with hyperactivation of this pathway, such as tuberous sclerosis complex and certain focal cortical dysplasias, other studies have reported no protective effect in different epilepsy models. For example, Gericke et al. [30] demonstrated that selective inhibition of mTORC1/2 or PI3K/mTORC1/2 signaling did not prevent or modify epilepsy in the intrahippocampal kainate mouse model. These findings suggest that the efficacy of mTOR-targeting interventions may depend on epilepsy etiology, disease stage, or cell-type-specific mechanisms, which could explain the lack of effect observed in our kindling experiments. Interestingly, previous studies have found that other drugs, such as anisomycin, inhibit protein synthesis, having an anti-epileptogenic effect when administered prior to kindling [31,32]. However, these drugs do not have an anti-convulsant effect when administered to kindled animals, which is consistent with the results presented here, as our animals received treatments after stimulation and after kindling had begun.

It is a valid concern that any intervention targeting memory consolidation could pose long-term cognitive risks, and mTOR is critical in several cellular processes. However, the therapeutic strategy explored here, with the treatment administered only within the brief consolidation window immediately following a seizure, was intended to selectively interfere only with the recently active seizure-related neural patterns. Additionally, a derivative of rapamycin has been approved by the American Food and Drug Administration for its use as a treatment for seizures in tuberous sclerosis patients following the success of clinical trials [33].

Following the advancement of the animal’s seizures, the animals were then subjected to an additional experiment that tested the effects of isoflurane on the persistence of epileptogenesis. Isoflurane was chosen due to its properties that potentiate GABAergic activity [34] and, thus, reduce global neuronal activity. The animals were kindled with electrical stimulation, like in the rapamycin experiment, followed by five minutes in an induction chamber filled with 2.5% isoflurane dissolved in oxygen. It was found that isoflurane did not affect changes induced by epileptogenesis, since the experimental animals continued to display advanced seizures following the treatments. However, anesthesia and other treatments have been shown to have an anti-epileptogenic effect when administered very early in the development of other models of epilepsy [35,36,37]. Thus, it is possible that the treatments applied in our study had no effect since they had started after the animals displayed intermediate seizures. Treating the animals at earlier stages could be more likely to disrupt memory consolidation processes and the progression of seizures, analogously, as it is easier to forget less established memories.

Given the results of this experiment, future studies should test interventions at earlier stages to clarify these mechanisms and their therapeutic implications for epileptogenesis. There are also other limitations of our study. For example, using recordings from single neurons [38] instead of LFP could provide more direct information about the effect of treatment on neuronal reactivation patterns, which are associated with memory consolidation [39]. Using histological analyses, like measuring cell density [40] or neuronal branching patterns [41], could also provide more details on the effect of rapamycin on epilepsy. Moreover, using machine learning techniques for analyses of animal behavior [42,43,44] could reveal more nuanced changes in behavioral seizures that human observers can miss with the standard Racine scale measure [45]. It would also be interesting to test other models of epilepsy, like the pentylenetetrazol kindling model. Nevertheless, the results presented here provide new insights into differences between epileptogenesis and memory processes, which could help researchers and clinicians in designing novel treatments for epilepsy.

## 4. Materials and Methods

### 4.1. Animals

All procedures were approved by the University of Lethbridge’s Animal Welfare Committee (AWC) and conducted in accordance with guidelines in the Canadian Council on Animal Care (CCAC). This study adheres to the ARRIVE (Animal Research: Reporting of In Vivo Experiments) guidelines for comprehensive reporting of animal research. Eighteen C57BL/6J mice aged 3–6 months, obtained from the Jackson Laboratories, Bar Harbor, Maine, were utilized for the experiments described in this study. The mice were housed within the vivarium at the University of Lethbridge in single cages (34.3 cm × 29.2 cm × 15.5 cm) lined with paper for enrichment, on a 12 h light/12 h dark cycle (700–1900 h) and had access to food and water ad libitum.

The rapamycin experiment consisted of a rapamycin-treated group (*n* = 6) and a saline-treated group (*n* = 6); both groups comprised 3 males and 3 females. After the rapamycin experiments were completed, the surviving animals were treated with isoflurane. The isoflurane experiment consisted of an isoflurane-treated group (*n* = 4) and a non-treated group (*n* = 6); the isoflurane-treated group comprised 2 males and 2 females, whereas the non-treated group comprised 3 males and 3 females. The isoflurane experiment utilized all animals from both the rapamycin- and saline-treated groups from the rapamycin experiment. However, because two animals died from their seizures at the end of the rapamycin experiment, the number of animals in the isoflurane experiments was smaller. It is unlikely that isoflurane had additive or synergistic effects with rapamycin, as the half-life of rapamycin is ~78 h [46], and the isoflurane experiments began >10 days after the last rapamycin injection; however, given mTOR’s involvement in multiple signaling pathways, indirect downstream effects of rapamycin may persist beyond its half-life and cannot be fully excluded. Following all experiments, the animals were euthanized with euthanasol (300 mg/kg), administered intraperitoneally, prior to decapitation and brain extraction.

### 4.2. Surgery

Surgeries were completed in groups of 6 on surgery days. The first surgery started in the morning (9 a.m.) with the final one in the late afternoon (2 p.m.). The animals had received an analgesic treatment of buprenorphine (0.075 mg/kg), administered subcutaneously 30 min pre-operatively. The animals were then anesthetized with 2.5% isoflurane dissolved in oxygen and received a local anesthetic of lidocaine (7 mg/kg) administered to the incision site. Bipolar electrodes (wire: A-M systems, Inc., 791400; pins; A-M systems, Inc., 520100) (Carlsborg, WA, USA) used for both recording and stimulation were implanted in the left basolateral amygdala (BLA) (M/L: −2.8, A/P: −1.3, D/V: −4.6; bregma was identified and used as a reference), with an additional unipolar ground electrode implanted posterior to lambda. Pins that were soldered to the opposite ends of the electrodes were then carefully loaded into a 6-channel electrode pedestal (plastics1) and then positioned so that the pins protruding from the pedestal were placed on the surface of the skull. The electrodes and pedestal were then secured with a base layer of Metabond, followed by several layers of dental acrylic that surrounded the pedestal. Surgery was completed once the dental acrylic dried. The animals were given seven days to recover and received post-operative care for a minimum of three days, during which the animals received analgesic treatment of Metacam (7.5 mg/kg, s.c.) and an antibiotic treatment of Baytril (5 mg/kg, s.c.) every 24 h.

### 4.3. Recording

The local field potential (LFP) from the BLA was recorded with a Digital Lynx SX acquisition system, and videos of the animal’s behavior were recorded with a Raspberry Pi model 4 B. The Raspberry Pi and Digital Lynx SX were synchronized by programming the Raspberry Pi to output a pulse corresponding to each video frame to the Digital Lynx SX. Additionally, the Raspberry Pi also outputs a pulse corresponding to each stimulus. For recordings, animals were placed in a cage. The cage was equipped with two LED lights to provide a visual stimulus and with two speakers for auditory stimulation. The roof of the chamber was equipped with a camera (RasPicamera). A tether that connects to the animal’s head cap was fed through a hole in the cage.

### 4.4. Kindling and Sensory Stimulation

A one-second 50 Hz biphasic train of square wave pulses delivered directly to the BLA was used to generate seizures. The stimulus was generated by programming a Master-8 pulse generator (A.M.P.I.) to output a train of 50 one msec square wave pulses every 20 ms over 1 s, with each pulse triggering an additional square wave pulse that was output 1 ms following the initial pulse [47]. The pulse was output by the Master-8 to the stimulus isolator unit A365 (SIU) (WPI: SYS-A365D) that isolated it from ground, converted it to a constant current, and made biphasic by reversing the polarity of the triggered pulse. To allow for both recording and stimulation from the same electrode in BLA, a relay was used. The Raspberry Pi controlled the delivery of the stimuli.

Before kindling stimulation, the sensory stimuli were presented as follows: one second auditory tone (110–130 dB; 12 kHz), one second pause, one second LED flash (white). This was followed by a four-second pause, then relay opening, a one-second pause, electrical stimulation, a one-second pause, and finally, the relay closing. The sensory stimuli were presented before kindling to test the effects of the treatments on memory processes. It was shown previously that in such experimental designs, animals develop freezing behavior in response to the sensory stimuli before kindling [48], similar to classical fear conditioning. Therefore, this design allowed us to measure the effect of treatments both on seizure intensity and on memory.

### 4.5. Experimental Timelines

**Figure 3:**
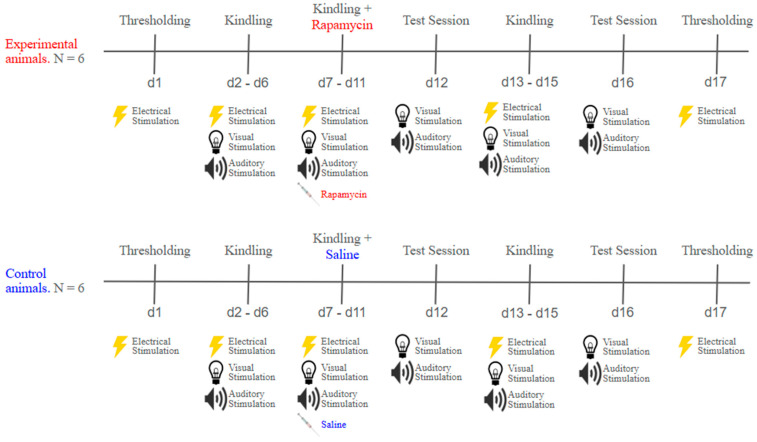
Timeline for rapamycin experiment. The timeline displays the order and the approximate days for each experimental phase. (**Top**) The experimental animals were subjected to several kindling sessions, in which visual and auditory stimuli were paired with electrical stimulation to induce seizures. Once the animals displayed sufficient seizures, they received an injection of rapamycin before being placed back into their home cage. (**Bottom**) The control animals were subjected to a similar protocol as the experimental animals; however, they received an injection of saline instead of rapamycin. In both groups, follow-up test sessions and thresholding were conducted to test the effects of rapamycin on fear learning and sensitization to the convulsive stimulus, respectively.

**Figure 4:**
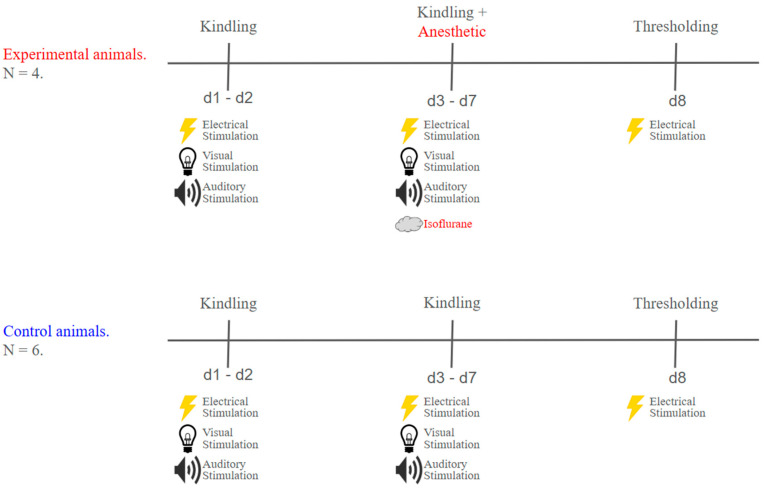
Timeline for the isoflurane experiment. The timeline displays the order and the approximate days for each experimental phase. (**Top**) The experimental animals were subjected to two kindling sessions, in which visual and auditory stimuli were paired with electrical stimulation to induce seizures. In the sessions that followed, after kindling, the animals were placed in an induction chamber filled with 2.5% isoflurane dissolved in oxygen. In the last session, the animals were rethresholded. (**Bottom**) The control animals followed a similar protocol as the experimental animals, except that they were immediately placed back into their home cage following kindling.

The rapamycin and isoflurane experiments were divided into 3 main phases: thresholding, kindling, and treatment, as described in the following sections and illustrated in Figure 3 and Figure 4. The experimental and control groups underwent sessions on alternating days.

#### 4.5.1. Thresholding

Seven days after surgery, and before kindling began, the minimum current to induce an after-discharge (AD) was determined for each animal. The sensory stimuli were absent for all thresholding sessions, and every animal received electrical stimulation starting at a current of 50 μA. Following stimulation, the EEG was monitored for an AD. If no AD was present, the current was increased by 50 μA and reapplied in intervals of 5 min until one was elicited. Once present, the suprathreshold was determined by adding 100 µA to the current that elicited the AD [19].

#### 4.5.2. Kindling

During the kindling phase [49], each animal was presented with the sensory stimuli, followed by the electrical stimulation, as described above. Animals were kindled once every second day (~48 h interval between kindling sessions). For each animal, a current equal to the suprathreshold was used. Baseline LFP activity was recorded for at least three minutes before stimulation was applied. In addition, seven minutes of LFP were recorded following stimulation. Kindling was applied over multiple days to progressively increase the strength and duration of a seizure, as described previously [14,19,48]. The rapamycin treatment was started when a stage 3 on the Racine scale was observed [45].

#### 4.5.3. Rapamycin Treatment

The rapamycin treatment consisted of five sessions, where, within 10 min after kindling, each animal received an intraperitoneal injection of either rapamycin (40 mg/kg) or saline for the control group. The rapamycin was prepared daily by dissolving the drug in a stock solution of 5% ethanol, 4% PEG 400, and 4% Tween 80 in distilled water that was no older than one week [20]. The control animals had received an injection of saline with a volume equal to that of rapamycin. After 48 h since the last injection, the animals were exposed to sensory stimuli in the absence of electrical stimulus (test sessions).

#### 4.5.4. Isoflurane Treatment

The isoflurane treatment also consisted of five sessions, where, within 10 min after kindling, the experimental animals were anesthetized with isoflurane (slow induction to 2.5% in oxygen) in an induction chamber for five minutes, while the control animals were returned to their home cages. The animals were kindled with new suprathreshold currents determined during the thresholding sessions prior to the first isoflurane experiment.

### 4.6. Analysis

#### 4.6.1. Duration of Electrographic Seizure

To determine seizure duration, a band-pass filter between 10 and 60 Hz was applied to the LFP signal from the BLA. The baseline consisted of the LFP signal recorded 1 min before the onset of the electrical stimulus, and a threshold was then set by calculating the standard deviation of the baseline activity and multiplying it by 5. Using the find peaks function in MATLAB (version R2023a), the ictal peaks above that threshold were detected within 2 min after kindling stimulation. The time of the final peak above the threshold was considered the seizure offset. Seizure duration was then calculated by taking the difference between the kindling onset and the seizure offset. All seizures were visually inspected to ensure the accuracy of this method.

#### 4.6.2. Freezing Duration

Kindling stimulation was preceded by sensory cues: visual and auditory stimulation. In this paradigm, it was shown that animals develop a post-cue freezing behavior that precedes electrical stimulation [48]. Freezing duration was determined by calculating the percentage of time that an animal did not move during the 7-s period in between the presentation of the auditory and electrical stimuli, as described in Das et al. [48]. In short, the video frames were converted into binarized images (black and white), and each frame was subtracted from the previous frame to quantify the animal’s movement. The percentage of time with movement below the predefined threshold was defined as freezing duration.

#### 4.6.3. Statistics

An equal number of sessions were analyzed for each animal that survived in all experiments; however, because it was ensured that the animals started treatment at roughly stage 3 of the Racine scale, several animals, particularly in the control group, required additional kindling sessions. A two-way analysis of variance (ANOVA) was conducted for both the rapamycin and isoflurane experiments to determine any significant effects of treatments and sessions on seizure duration, behavioral seizure, and post-cue freezing. A *t*-test was used to determine whether there were any significant differences between the two groups in individual sessions. A three-way ANOVA was conducted to determine whether there were significant effects of the treatments, kindling, and session on the swim latencies during training of the MWT. A two-way ANOVA was utilized to determine whether there were significant effects of the kindling and treatments on spatial memory retention during the MWT probe. *p* < 0.05 was considered statistically significant.

## Data Availability

The datasets used and/or analyzed during the current study are available from the corresponding author on reasonable request. Data from neurophysiological recordings is also publicly available at our website https://people.uleth.ca/~luczak/Epil_Data/, accessed on 20 August 2020.
